# CircRNA_100395 inhibits cell proliferation and metastasis in ovarian cancer via regulating miR-1228/p53/epithelial-mesenchymal transition (EMT) axis

**DOI:** 10.7150/jca.35041

**Published:** 2020-01-01

**Authors:** Xian Li, Shuihua Lin, Zhifeng Mo, Jinxing Jiang, Haifeng Tang, Cailin Wu, Jian Song

**Affiliations:** 1 Department of Obstetrics and Gynaecology, The University of Hong Kong - Shenzhen Hospital, Shenzhen, Guangdong, China.; 2 Department of Medical Imaging, The University of Hong Kong - Shenzhen Hospital, Shenzhen, Guangdong, China.; 3 Department of Emergency & Disaster Medical Center, The Seventh Affiliated Hospital, Sun Yat-sen University, Shenzhen, Guangdong, China.; 4 Cytotherapy Laboratory, Shenzhen People's Hospital, The Second Clinical Medical College of Jinan University, Shenzhen, Guangdong, China.; 5 Department of Obstetrics and Gynaecology, The University of Hong Kong - Shenzhen Hospital, Shenzhen, Guangdong, China.; 6 Department of Obstetrics and Gynaecology, The University of Hong Kong - Shenzhen Hospital, Shenzhen, Guangdong, China.; 7 Department of Obstetrics and Gynaecology, The University of Hong Kong - Shenzhen Hospital, Shenzhen, Guangdong, China.

**Keywords:** CircRNA_100395, Cell Growth, Metastasis, EMT, Ovarian Cancer.

## Abstract

**Purpose:** Circular RNAs (circRNAs) have been reported to regulate the incidence of tumor by regulating the transcriptional level and post-transcriptional level of tumor-related genes, and are significantly correlated with tumor metastasis and progression. CircRNA_100395 (circ_100395) has been reported to suppress lung cancer cell proliferation, and might act as an oncogene in deveopment of various cancers. However, the expression and function of circ_100395 in ovarian cancer has not been systematically researched.

**Methods:** The expression of circ_100395 in ovarian cancer tissues was detected by Real-time Quantitative polymerase chain reaction (RT-qPCR), while the relationship between circ_100395 expression and clinicopathological characteristics was further analyzed. After increasing the expression of circ_100395 by plasmid transfection in ovarian cancer cells, we further investigated the cell proliferation, invasion and migration by cell counting kit-8 (CCK-8), and Transwell assays. Epithelial-mesenchymal transition (EMT) pathway was also measured by western blotting. In addition, the relationship among circ_100395, miR-1228 and p53 in ovarian cancer, was explored by luciferase reporter assay.

**Results:** The expression of circ_100395 was found to be significantly down-regulated in ovarian cancer, while low expression of circ_100395 was highly correlated with the poor outcomes. In addition, upregulation of circ_100395 could significantly inhibit tumor growth, metastasis and EMT signaling pathway in ovarian cancer. Furthermore, the expression level of circ_100395 was negatively correlated with the expression of miR-1228, and with the addition of miR-1228 could reverse anti-cell proliferation effect induced by circ_100395 in ovarian cancer cells. In addition, p53 might be the key target of circ_100395 / miR-1228 axis in ovarian cancer.

**Conclusion:** CircRNA_100395 could inhibit cell growth and metastasis of ovarian cancer cells via regulating the miR-1228/p53/EMT axis.

## Introduction

Ovarian cancer is one of the most common malignant tumors worldwide, with 25,000 deaths annually. Most of the patients with ovarian cancer was diagnose at an advanced stage, and the treatment outcome of ovarian cancer is usually poor^1^-^4^. Therefore, in order to improve the survival rates of ovarian cancer patients, it is imperative to explore a novel bio-marker for early diagnosis and effectively treatment.

Circular RNAs (circRNAs), firstly identified in RNA virus and later observed in eukaryotic cells as an endogenous RNA splicing product, are a class of non-coding RNAs with covalently bonded circular structure, but without 5′ caps and 3′ tails^5^, ^6^. Thanks to the development of high-throughput sequencing, molecular biology techniques and bioinformatics, the expression patterns and diverse functions of circRNAs are being elucidated^7^, ^8^. They participated in multiple physiological and pathological processes through sponging miRNAs and other RNAs by base pairing, interacting with proteins by their three-dimensional structure with a high degree of tissue-specific expression^9^, ^10^, while increasing studies indicated that non-coding RNAs (ncRNAs) was closely related to the tumorigenesis^11^, ^12^.

Actually, increasing researches demonstrated that dysfunction of circRNAs played a significant role in the occurrence and progression of ovarian cancer, including circEXOC6B^13^, circN4BP2L2^13^, circ_0061140^14^ and circ-ITCH^15^, ^16^. Circ_100395, also called circ_0015278, was located at chromosome 1q25.1 and composed of four exons of KLHL20 mRNA. Besides, Chen et al. had confirmed that circ_100395 could suppress the lung cancer progression, and circ_100395 was also closely associated with the tumor cellular malignant phenotype^17^. However, whether circ_100395 functioned as an oncogene or tumor suppressor in ovarian cancer was still unclear. In addition, accumulating studies have shown that EMT was a significant mechanism in the metastasis of malignant tumor, and this signaling pathway could be regulate by circRNAs.

In the present research, the relative expression of circ_100395 in ovarian cancer tissues and cells was demonstrated, the potential function of circ_100395 was analyzed by the correlation analysis with the clinicopathological parameters of ovarian cancer patients. Then, the underlying mechanism that contributing to the down-regulation of circ_100395 in ovarian cancer cell lines was investigated. Furthermore, the effects of circ_100395 on cell growth, metastasis and the process of EMT, as well as the possible mechanism were explored via* in vitro* assays and bioinformatics.

## Materials and methods

### Patient tissue samples

Ovarian cancer and precancerous lesions were selected from 60 patients who had not been treated with radio- or chemical therapy, in the Shenzhen Hospital of Hong Kong University, between 2012 and 2014. The details of patient's enrollment are as the followings: 1. Age older than 18 and younger than 80 years; 2. Written informed consent; 3. Primary ovarian cancer and confirmed pathologically by veteran pathologists. In addition, the exclusion criterions are showed as below: 1. Patients with other malignant diseases; 2. Patients with previous neoadjuvant chemotherapy or radiotherapy. The medical ethics committee of Shenzhen Hospital of Hong Kong University approved the study. Furthermore, another cohort with ovarian cancer from Sun Yat-sen University, were also selected as validation cohort, while the medical ethics committee of Sun Yat-sen University approved the study.

### Cell culture

Five ovarian cancer cell lines (A2780, OV2008, SKOV3, IGROV1 and ES-2) and epithelial normal cell line, and were acquired from ATCC (Shanghai, China). All the cell lines were grown in Dulbecco's Modified Eagle Medium (Gibco, Grand Island, NY, USA) supplemented with 10% fetal bovine serum (FBS; Gibco) at 37℃ and 5% CO_2_.

### RNA transfection

Has-circ_100395 cDNA plasmid (pcDNA3.1) were firstly developed by GENESEED (Guangzhou, China). Following that, the lentiviruses expressing circ_100395 (OE- circ_100395) and the relative negative control (OE-Vector) were subsequently constructed by GENE (Shanghai, China). The ovarian cancer cells (SKOV3 and ES-2) were collected, seeded, then transfected with OE-circ_100395 or OE-Vector lentiviruses. To date, the multiplicity of infection [MOI] is 50 for SKOV3 cells, while 20 for ES-2cells. After the transfection for 48 hours, the SKOV3 and ES-2 cells were again cultured with free medium in the presence of polybrene for another 5 days. To date, the concentration of polybrene was 4μg/ml for SKOV3 cells, while 3μg/ml for ES-2cells. After that, the cells were collected and applied for study. To analyze the relationship of circ_100395, miR-1228 and p53, miR-1228 mimics and p53-specific cDNA plasmid (pcDNA3.1) were also developed by GENESEED and transfected with Lipofectamine 3000 (Invitrogen; USA). The sequences of circ_100395, miR-1228 and p53 were shown in** Table 1.**

### Quantitative real-time polymerase chain reaction (RT-qPCR)

Total RNA from the samples was extract by RNAiso Plus reagent (TaKaRa, Tokyo, Japan). Following that, RNA was reversed to cDNA with the PrimeScript RT Master Mix (TaKaRa). Then, the SYBR Premix Ex Taq II Kit (TaKaRa) was used to determine the expression level of circ_100395 and p53 through the StepOnePlus system (Applied Biosystems, CA, USA). The expression was calculated using the 2^-ΔΔCt^ method. To date, for the analysis of miR-1228 expression, RT primer mix (containing oligo-dT primer and random hextamers) was replaced with RT primers. The primers sequences are listed in **Table 1**.

### *In vitro* cell growth assay

To analyze the cell growth, cell counting kit 8 (CCK-8, Donjindo, Kumamoto, Japan) assay was performed. 100 μL medium containing 5×10^3^ ovarian cancer cells, were firstly plated into 96-well plates. Then, the CCK-8 was added to the wells, and OD value of the treated cells was assessed from one to four days.

### Cell migration assay

The transwell assays were performed to evaluate the cell migration and invasion abilities. Firstly, the serum-free McCoy's 5a Medium contained 5×10^4^ cells were supplemented into the upper compartments, while the bottom compartment with 500 μl Dulbecco's Modified Eagle Medium with 20% FBS. 24 hours later, the cotton-tipped swabs were applied to remove the non-migrating and non-invading cells, then the other cells on the membranes were fixed and stained with 0.5% crystal violet. Finally, the migrated and invaded cells were evaluated under the microscope.

### Western blotting

Firstly, the RIPA lysis buffer (Beyotime;Shanghai, China) was applied to extract the total protein from the cell precipitation, and its concentration was measured using BCA protein Assays Kit (Thermo Fisher Scientific; Shanghai, China). Equal amount of proteins (20 μg) was separated on SDS-PAGE gel, and then transferred onto polyvinylidene fluoride (PVDF) membranes. Subsequently, the membranes contained proteins were incubated with 5% BSA, the specific primary and secondary antibody respectively. Finally, the protein bands were visualized by GeneSnap using SynGene system.

### Dual-luciferase reporter assays

Based on the prediction provided by publicly available bioinformatic algorithms (Starbase), miR-1228 might be a downstream target for circ_100395. To address that issues, the 293T cells were plated in 24-well plates at the concentration of 5 × 10^4^ cells/well. Subsequently, cells were transfected with pmirGLO-circ_100395-WT or pmirGLO-circ_100395-MUT plasmid, with miR-1228 mimics or miR-1228 NC. After 48 hours, passive lysis buffer (Promega, Beijing, China) was applied to lyse the cells, and the Dual-Luciferase Reporter Assay (Promega, Madison, WI, USA) was used to calculate the relative luciferase activity through normalizing the firefly luminescence to Renilla luminescence. To date, based on the prediction provided by publicly available bioinformatic algorithms (Starbase), p53 might be a downstream target for miR-1228. To address that issues, sample method was applied, following by 293T cells were transfected with pmirGLO-p53-WT or pmirGLO-p53-MUT plasmid, with miR-1228 mimics or miR-1228 NC.

### Statistics

All data in this study was shown as mean ± standard deviation (SD). The GraphPad Prism 7.0 (GraphPad Software, Inc, CA, USA) was applied to evaluate these data. In addition, all statistical analysis were performed with the two-tailed Student's t-test, analysis of variance (ANOVA) or the Pearson test. Moreover, both of Kaplan-Meier method and log-rank test were performed to analyze the survival data. When *P* < 0.05, significant difference was considered.

## Results

### Circ_100395 was down-regulated in ovarian cancer and its low-expression predicted poor prognosis

**Figure 1:**
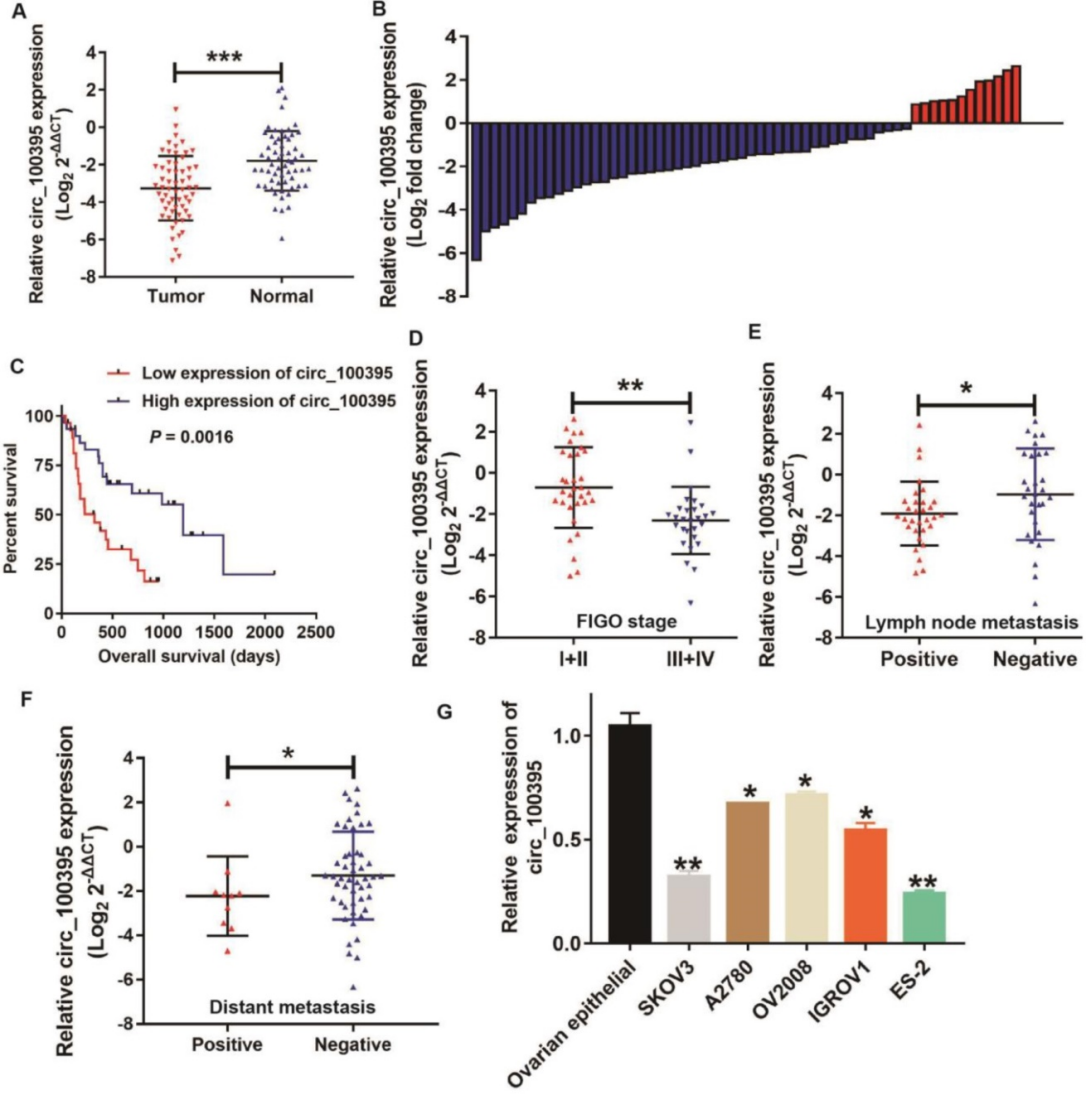
** The expression of circ_100395 in ovarian cancer patients and the relationship with patients' clinicopathological parameters.** (A) circ_100395 was significantly down-expressed in ovarian cancer tissues than in adjacent normal tissues evaluated by RT-qPCR. (B) The relative circ_100395 expression was down-regulated in 80% (48/60) patients with ovarian cancer revealed as the form of Log2 (T/N). (C) The correlation between the expression of circ_100395 and overall survival times of patients with ovarian cancer. All experiments were repeated at least three time. (D) The correlation between the expression of circ_100395 and FIGO stage. (E) The correlation between circ_100395 expression and lymph node metastasis. (F) The correlation between circ_100395 and distant metastasis. (G) The expression of circ_100395 is down-regulated in ovarian cancer cell lines (SKOV3, A2780, OV2008, IGROV1 and ES-2) compared with that in normal ovarian epithelial normal cell line. *P<0.05, **P<0.01, ***P<0.001.

In order to detect the relationship between the expression level of circ_100395 and the clinicopathological characteristics of ovarian cancer patients, RT-qPCR was performed to analyze the circ_100395 expression in ovarian cancer tissues and adjacent normal tissues. The results displayed that circ_100395 was significantly down-expressed in ovarian cancer (Figure 1A). Moreover, low-expression of circ_100395 accounted for 80% (48/60) of ovarian cancer specimens (Figure 1B). Meanwhile, another validation cohort containing 60 patients from another center, was also applied to detected the expression of circ_100395 in ovarian cancer. Interestingly, the qPCR results showed that circ_100395 was also inhibited in ovarian cancer (Figure S1), Consistently, the low-expression of circ_100395 accounted for 75% (45/60) of ovarian cancer specimens (Figure S2). Consequently, our results indicated that circ_100395 did being inhibited in patients with ovarian cancer. To date, our results also revealed that ovarian cancer patients with lower expression level of circ_100395 were more likely to have shorter overall survival time (Figure 1C). These results showed circ_100395 was linked to the poor clinical outcomes of ovarian cancer. To further explore the relationship between circ_100395 and clinicopathological characteristics, we divided the tissue samples into two groups (high expression group and low expression group) according to the median of the relative circ_100395 expression. From the correlation analyses, the expression of circ_100395 was closely related to the lymphatic metastasis (*P*=0.004), distant metastasis (*P*=0.038) and FIGO stage (*P*=0.000) (Table 2). Consistently, our data also showed that the ovarian cancer patients with lower expression of circ_100395 were more likely to have high FIGO stage (Figure 1D), lymph node metastasis (Figure 1E) and distant metastasis (Figure 1F). Together, these results revealed that circ_100395 was down-regulated in ovarian cancer tissues, and its expression was highly correlated with the poor oncological outcomes.

### Overexpression of circ_100395 suppresses the proliferation in ovarian cancer cells

**Figure 2:**
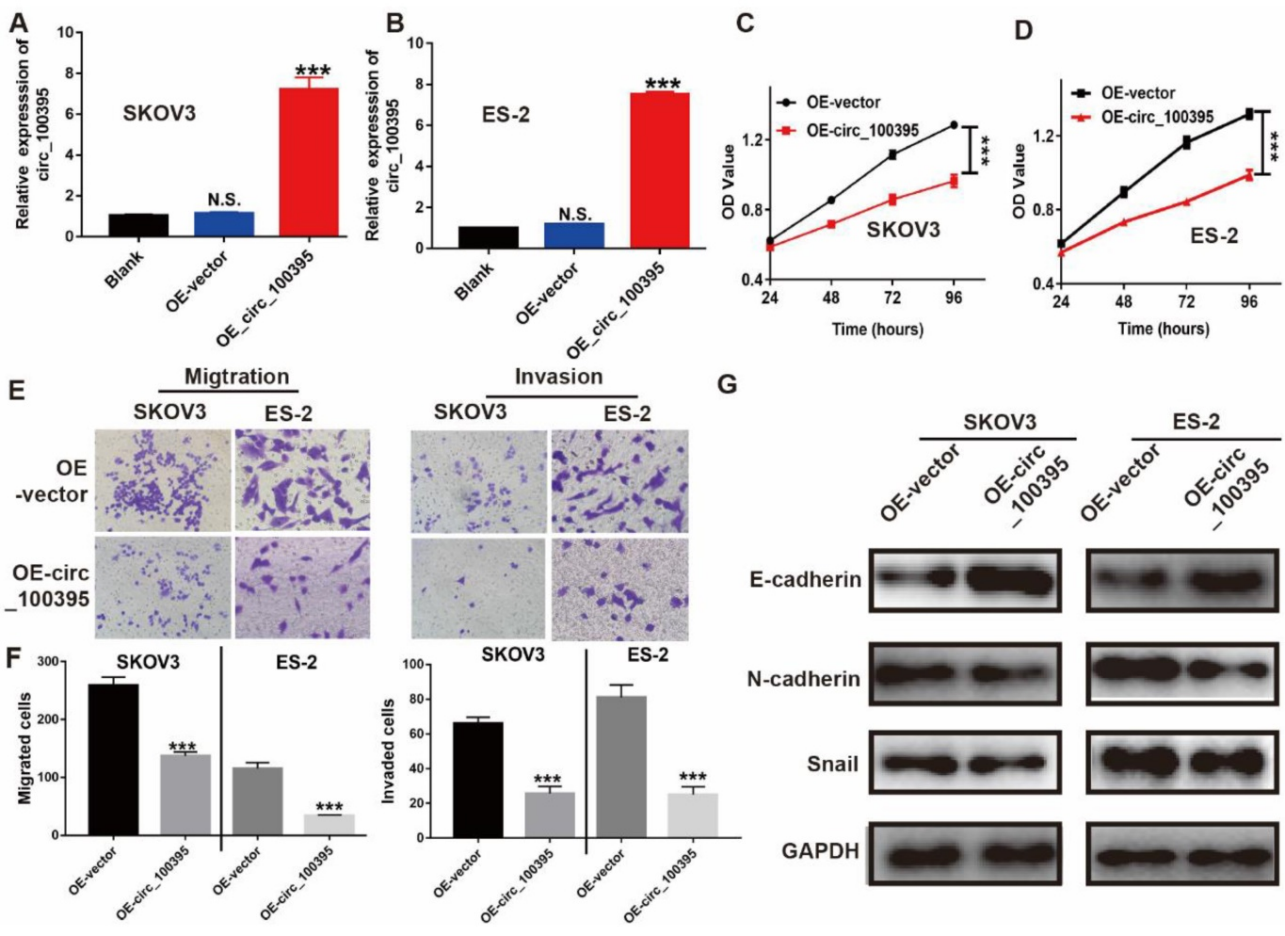
**Overexpression of circ_100395 suppresses the proliferation, migration, invasion and process of epithelial-mesenchymal transition** (**EMT**) **in ovarian cancer cells.** (A & B) The overexpression efficiency of circ_100395 by a lentivirus-based method in SKOV3 and ES-2 cell lines. (C & D) The effect of up-regulated circ_100395 significantly inhibit the proliferation of SKOV3 and ES-2 cells *in vitro* using CCK-8 assay. (E & F) The overexpression of circ_100395 significantly reduced the number of migrated cells in SKOV3 and ES-2 cell lines, measured by Transwell assay. (G) After the overexpression of circ_100395, the expression of the epithelial marker, E-cadherin was significantly upregulated, while the mesenchymal markers, N-cadherin and Snail were dramatically downregulated in both SKOV3 and ES-2 cell lines. *** *P* < 0.001, N.S. means no significant difference.

To further clarify the biological significance of circ_100395 in ovarian cancer, we firstly increased the expression of circ_100395 in ovarian cancer cell lines. In our study, RT-qPCR results showed that, compared with that in the normal ovarian epithelial normal cell line, the endogenous expression level of circ_100395 in ovarian cancer cell lines (SKOV3, A2780, OV2008, IGROV1 and ES-2) was significantly down-regulated, while SKOV3 and ES-2 cell lines showing the highest expression (Figure 1G). Then, we established circ_100395-overexpressed (OE- circ_100395) SKOV3 and ES-2 cell lines via lentivirus transfection. The RT-qPCR results showed that the expression level of circ_100395 was not changed in the negative control group (OE-Vector) compared with that in the blank group (without treatment). The transfection of pcDNA3.1-plasmids greatly increased the expression of circ_100395 in SKOV3 (Figure 2A) and ES-2 (Figure 2B) cell lines. Subsequently, CCK-8 assays were performed to evaluate the *in vitro* proliferation abilities of these stable OE- circ_100395 cell lines. Our results showed that the up-regulation of circ_100395 could effectively suppress the proliferation of SKOV3 and ES-2 cells (Figure 2C & 2D). Therefore, *in vitro* CCK-8 assay suggested that circ_100395 could inhibit cell proliferation of ovarian cancer cell lines.

### Overexpression of circ_100395 suppresses the migration, invasion and process of epithelial-mesenchymal transition (EMT) in ovarian cancer cells

**Figure 3:**
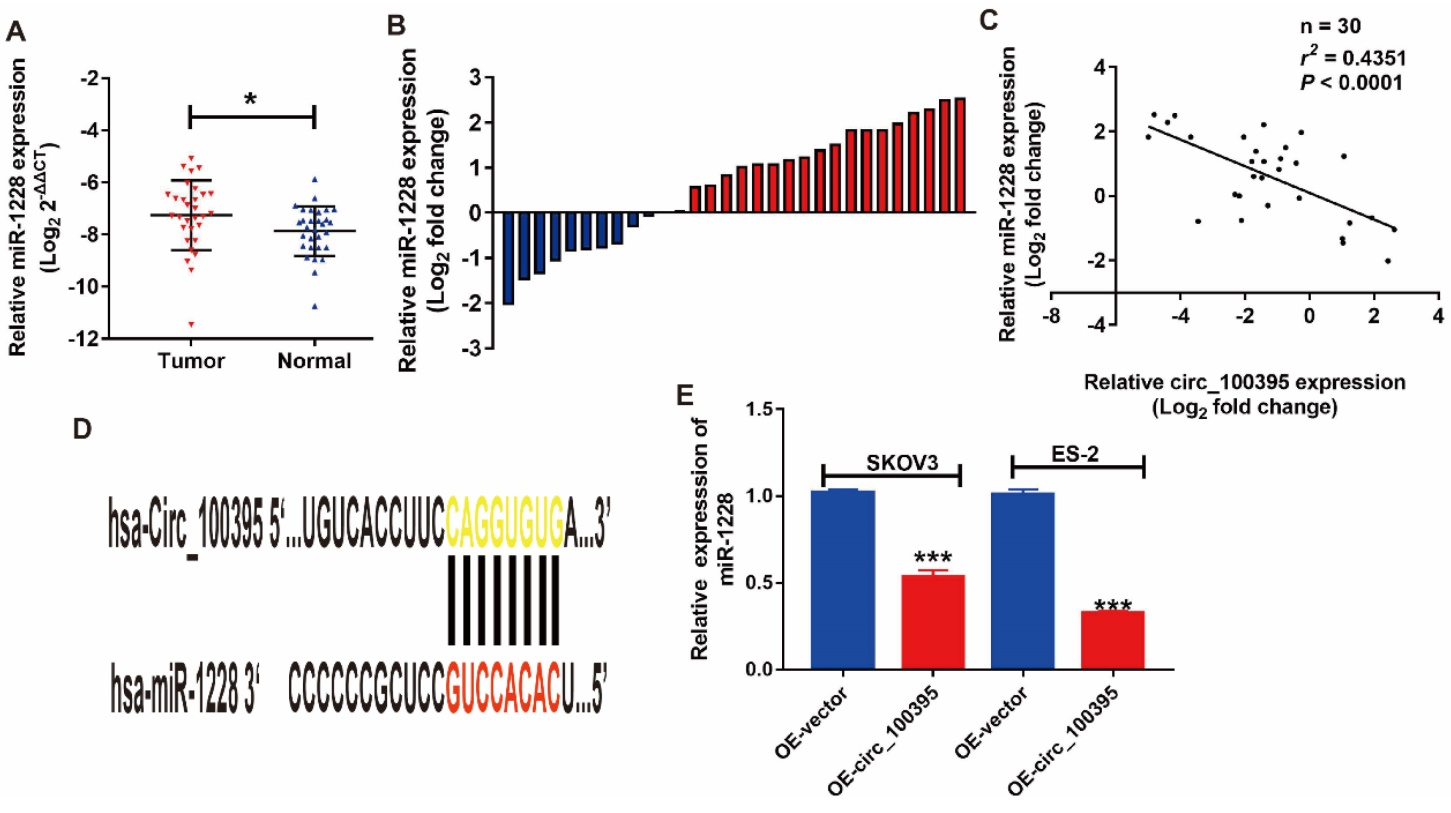
** miR-1228 is a direct target of circ_100395 in ovarian cancer.** miR-1228 was significantly overexpressed in ovarian cancer tissues than in adjacent normal tissues evaluated by RT-qPCR. (B) The relative miR-1228 expression was up-regulated in 66.7% (20/30) patients with ovarian cancer revealed as the form of Log2 (T/N). (C) Correlation between circ_100395 and miR-1228 at mRNA level in 30 paired human ovarian cancer tissues. (D) The predicted 3ʹUTR binding regions circ_100395 on miR-1228. (E) The expression level of miR-1228 in circ_100395-overexpressed ovarian cancer cells. *P<0.05, ***P<0.001.

Circ_100395 might be involved in regulating ovarian cancer metastasis, since the expression of circ_100395 was highly associated with the N stage and distant metastasis. Consequently, Transwell assays were applied to evaluate the effect of circ_100395 on cellular migration and invasion capabilities. After overexpression of circ_100395, the number of migrated and invasive cells were significantly reduced in SKOV3 and ES-2 cell lines, from the images (Figure 3E). Similarly, statistical data showed the same results in SKOV3 and ES-2 cell lines (Figure 3F).

To study the potential mechanism of circ_100395 on anti-tumor abilities, the expression of EMT-related markers were detected by western blotting. After overexpression of circ_100395, E-cadherin (epithelial marker) was upregulated, while N-cadherin and Snail (mesenchymal markers), were downregulated in both SKOV3 and ES-2 cell lines (Figure 3G)**.** Therefore, we concluded that circ_100395 inhibit proliferation, metastasis and EMT signaling pathway in ovarian cancer.

### MiR-1228 might be the downstream of circ_100395 on ovarian cancer

Based on the prediction provided by publicly available bioinformatic algorithms (Starbase), miR-1228 might be a downstream target for circ_100395. To address that issues, we evaluated the expression pattern of miR-1228 in ovarian cancer patients. From the RT-qPCR results, miR-1228 was significantly overexpressed in ovarian cancer tissues than in adjacent normal tissues (Figure 3A). Consistently, miR-1228 was overexpressed in 66.7% (20/30) patients with ovarian cancer (Figure 3B). The correlation analysis showed that the expression level of miR-1228 was negatively correlated with the expression of circ_100395 (Figure 3C, *r*=-0.4351, *P<*0.0001). In the meantime, another validation cohort containing 30 patients from another center, was also applied to detected the expression of miR-1228 in ovarian cancer. Surprisingly, our validation data also showed that miR-1228 was significantly overexpressed in ovarian cancer tissues (Figure S3), as miR-1228 was overexpressed in 76.7% (23/30) patients with ovarian cancer (Figure S4). Furthermore, while expression level of miR-1228 was negatively correlated with the expression of circ_100395 (Figure S5, *r*=-0.5197, *P<*0.0001). Consequently, our results indicated that miR-1228 did being highly expressed in patients with ovarian cancer, and its negative relationship with circ_100395 indicated that miR-1228 might be the downstream of circ_100395. In addition, we predicted that circ_100395 could bind to the 3ʹUTR regions of miR-1228 (Figure 3D). Consistent with the previous results, the expression of miR-1228 was reduced by 40% in circ_100395-overexpressed SKOV3 cells, while 70% in circ_100395-overexpressed ES-2 cells (Figure 3E). These results showed circ_100395 and miR-1228 was highly associated in ovarian cancer.

### Up-regulation of miR-1228 reverses the anti-cell proliferation effect induced by overexpression of circ_100395 in ovarian cancer cells

**Figure 4:**
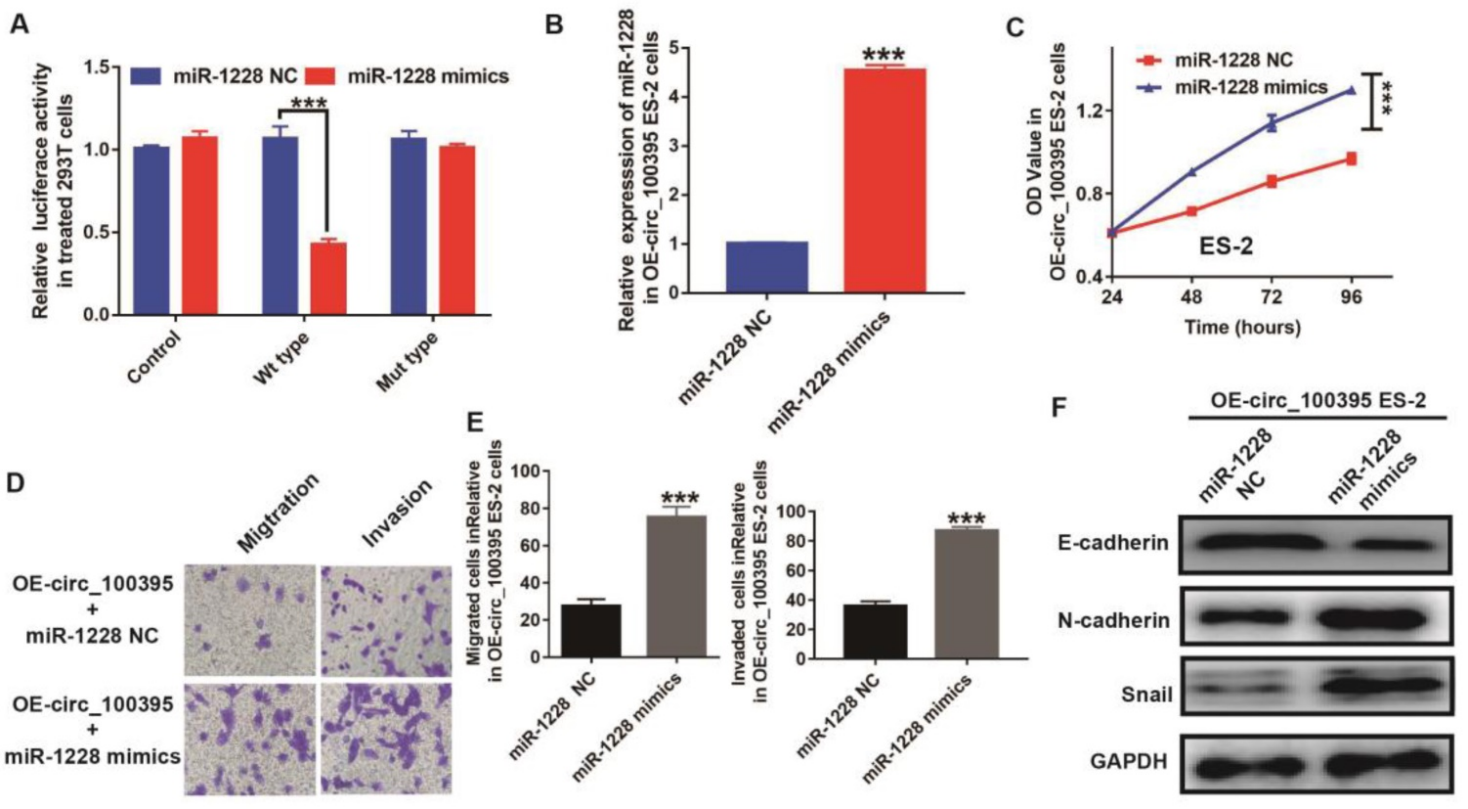
** Up-regulation of miR-1228 reverses the anti-cell proliferation effect on circ_100395 overexpressed (OE- miR-216a-5p) ovarian cancer cells.** (A) The relative luciferase activity in 293 T cells after co-transfection with pmirGLO- circ_100395-WT or pmirGLO- circ_100395-MUT, along with miR-1228 mimics or miR-1228 NC. (B) The overexpression efficiency of miR-1228, after transfected with miR-1228 mimics or relative negative control (miR-1228 NC) by Lipo3000 in circ_100395-overexpressed ES-2 cell lines. (C) The up-regulated miR-1228 significantly inhibited the proliferation of circ_100395-overexpressed ES-2 cells *in vitro* using CCK-8 assay. (D & E) The overexpression of miR-1228 significantly increased the number of migrated and invaded cells in circ_100395-overexpressed ES-2 cells, measured by Transwell assay. (F) After the overexpression of miR-1228, the expression of the epithelial marker, E-cadherin was significantly reduced, while the mesenchymal markers, N-cadherin and Snail were dramatically increased in circ_100395-overexpressed ES-2 cell lines. *** *P* < 0.001

To further explore the relationship between the expression of miR-1228 and circ_100395, we constructed the circ_100395 mutant type 293T cells. From the luciferase reporter assay, the luciferase activity was significantly lower in pmirGLO-circ_100395-WT cells treated with miR-1228 mimics, compared to the control and pmirGLO-circ_100395-MT groups (Figure 4A). This data showed miR-1228 might be the downstream target of circ_100395. To explore the role of miR-1228 during circ_100395 regulating ovarian cancer cells, we tried to upregulate the expression of miR-1228 in ES-2 cells, as circ_100395-overexpressed ES-2 cells showed higher efficiency on inhibiting the expression of miR-1228. From the RT-qPCR results, miR-1228 mimics could dramatically upregulate the expression of miR-1228 (4 folds) in circ_100395-overexpressed ES-2 cells, indicating that the miR-1228-overexpressed cells had been successfully constructed(Figure 4B). Interestingly, the results of CCK-8 showed that the overexpression of miR-1228 increased the cell growth in circ_100395-overexpressed ES-2 cells (Figure 4C). In addition, the anti-cell migration and invasion effect induced by circ_100395, could be reversed by miR-1228 mimics (Figure 4D & E). Furthermore, after the addition of miR-1228 mimics, the expression of the epithelial marker, E-cadherin was significantly reduced, while the mesenchymal markers, N-cadherin and Snail were dramatically increased in circ_100395-overexpressed ES-2 cells (Figure 4F).

### Circ_100395 suppresses the proliferation, migration, invasion and EMT in ovarian cancer cells, by sponging to miR-1228 and regulating p53 in ovarian cancer

**Figure 5:**
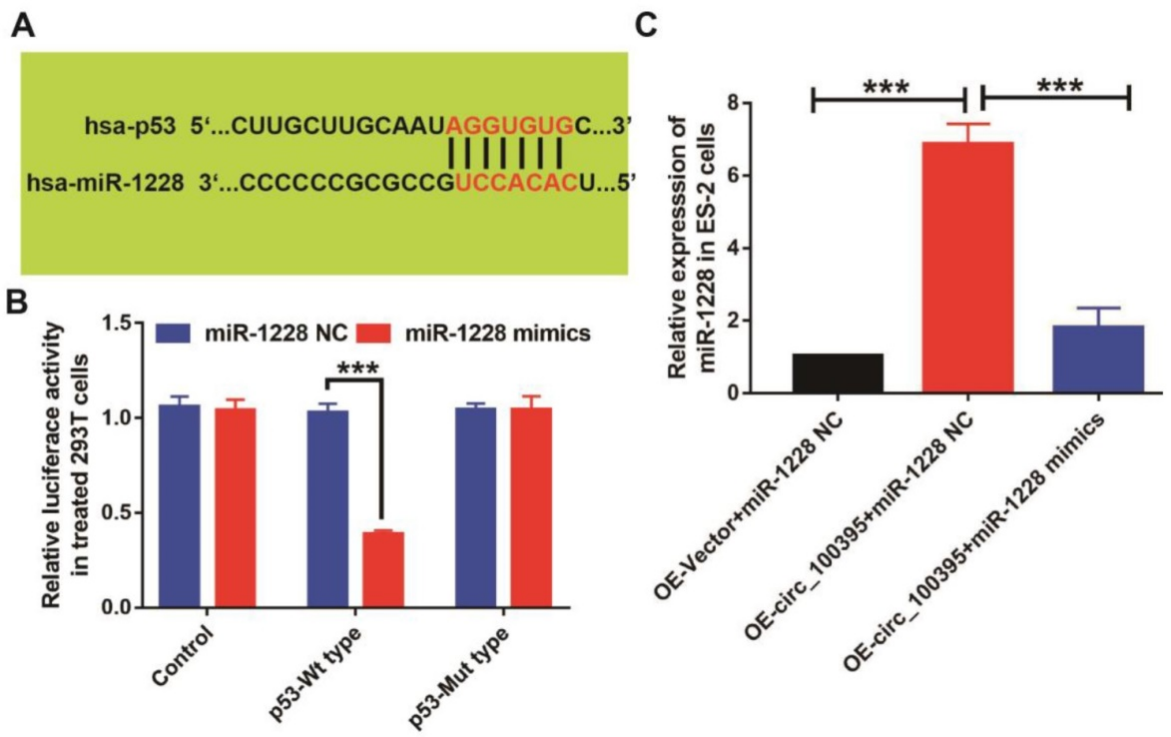
** p53 might be the key target of circ_100395/miR-1228 axis in ovarian cancer.** (A) The predicted 3ʹUTR binding regions miR-1228 on p53. (B) The relative luciferase activity in 293 T cells after co-transfection with pmirGLO-p53-WT or pmirGLO-p53-MUT, along with miR-1228 mimics or miR-1228 NC. (C) The expression level of p53 in circ_100395 or miR-1228 up-regulated ovarian cancer cells. *** *P* < 0.001

To further explore the downstream target of circ_100395/miR-1228 axis in ovarian cancer, we predicted target genes of miR-1228 from TargetScan and miRanda databases, while p53 was found to be the potential downstream targets, as the predicted 3ʹUTR binding regions miR-1228 on p53 was shown in Figure 5A. To explore their relationship, we constructed the p53 mutant type 293T cells. From the luciferase reporter assay, the luciferase activity was significantly lower in pmirGLO- p53-WT cells treated with miR-1228 mimics, compared to the control and pmirGLO-p53-MT groups (Figure 5B). We also detected the expression of p53 at mRNA levels in OE-circ_100395 ES-2 cells with or without miR-1228 mimics treatment. Interestingly, the expression of p53 was found to be significantly up-regulated in OE- circ_100395 ES-2 cells, but again reduced by miR-1228 mimics (Figure 5C). These results showed that p53 might be the downstream regulatory target of circ_100395/miR-1228 axis.

**Figure 6:**
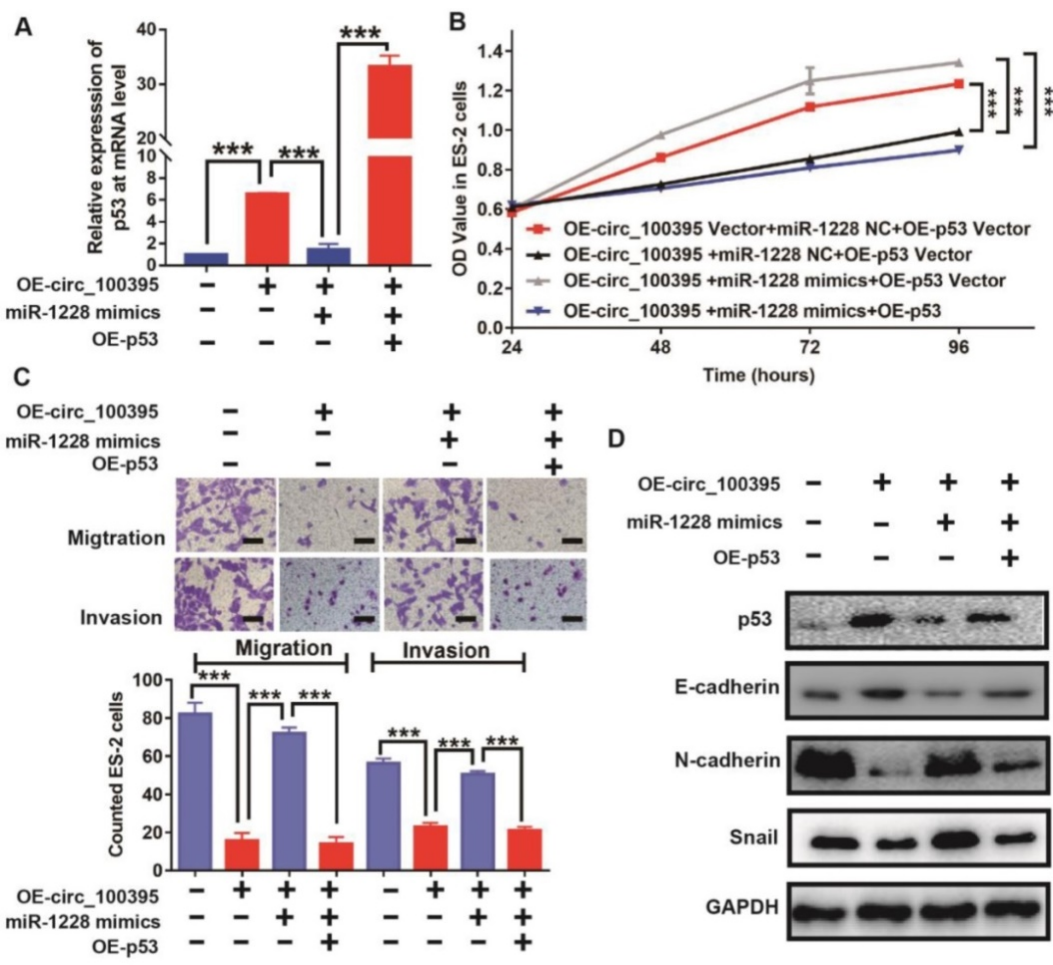
** circ_100395 suppresses the proliferation, migration, invasion and EMT in ovarian cancer cells, by sponging to miR-1228 and regulating p53 in ovarian cancer.** (A) The expression level of p53 in circ_100395 or miR-1228 or p53 up-regulated ovarian cancer cells. (B) The overexpression of circ_100395 or miR-1228 or p53 regulate the proliferation of ES-2 cells *in vitro* using CCK-8 assay. (C) The overexpression of circ_100395 or miR-1228 or p53 significantly regulated the number of migrated and invaded cells in ES-2 cells, measured by Transwell assay. (D) Overexpression of circ_100395 or miR-1228 or p53 dramatically changed the expression of the epithelial marker, E-cadherin, while the mesenchymal markers, N-cadherin and Snail were contrarily regulated. *** *P* < 0.001.

To further validate this hypothesis, we increased the expression of p53 in ES-2 cells, treated with OE-circ_100395 or OE-miR-1228. From the RT-qPCR results, p53 was upregulated in OE-circ_100395 cells, but reduced by the addition of miR-1228 mimics. Again, p53-specific pcDNA3.1-plasmid could greatly induce overexpression of p53 (OE-p53) in ES-2 cells (Figure 6A). Interestingly, the results of CCK-8 showed that the overexpression of miR-1228 increased the cell growth in circ_100395-overexpressed ES-2 cells, but again inhibited by OE-p53 (Figure 6B). Consistently, the anti-cell migration and invasion effect induced by circ_100395, was reversed by miR-1228. However, OE-p53 could rescue this effect, as less migration and invasion cells were observed from Transwell assays (Figure 6C). Furthermore, after the addition of p53, the expression of the epithelial marker, E-cadherin was significantly increased, while the mesenchymal markers, N-cadherin and Snail were dramatically reduced in OE-circ_100395 plus miR-1228 mimics ES-2 cells (Figure 6D). Consequently, these results showed that circ_100395 suppressed the proliferation, migration, invasion and EMT signaing pathway in ovarian cancer cells via sponging to miR-1228 and regulating the expression of p53 in ovarian cancer.

## Discussion

Growing women are diagnosed as ovarian cancer, and its mortality rate ranks first among gynecological tumors. In recent year, the immunotherapy and molecular target therapy had made a big through, however, the 5-year survival rate of ovarian cancer was still unsatisfactory. Therefore, identifying a novel bio-marker for ovarian cancer is an urgent issue^1^, ^2^, ^11^.

Accumulating evidences indicated that circRNAs were closely related to the occurrence and progression of tumors, and which played important roles as cancer promoters or suppressors. For example, circ-001569 is reported to promote tumorigenesis and invasion of hepatocellular carcinoma cells^18^, and circ_0066444 suppresses proliferation and metastasis of gastric cancer^19^. Among all, low expression of circ_100395 had already been identified in lung cancer^17^. However, little is known about circ_100395 in the regulation of ovarian cancer.

In the present study, we explored the biological value of circ_100395, following by analyzing the expression pattern of circ_100395 in ovarian cancer. Circ_100395 expression levels in ovarian cancer tissues were markedly lower than those in the corresponding noncancerous tissues. Similarly, another validation cohort from Sun Yat-sen University also showed that circ_100395 was downregulated in patients with ovarian cancer specimens, indicating that circ_100395 might serve as cancer suppressor in ovarian cancer. Moreover, our data also showed that ovarian cancer patients with lower expression of circ_100395 were more likely to have advanced FIGO stage, lymph node metastasis, distant metastasis and shorter survival time. To date, Chen's study also reported that circ_0061140 was upregulated in ovarian cancer cells, in turn, promoting cell proliferation, migration, and the EMT^14^. Luo's research found a broad down-regulation of circ-ITCH in ovarian cancer tissues and cells, which correlates with a worse prognosis in ovarian cancer patients^15^, while circ-ITCH might play a novel tumor suppressive role in the malignant progression of ovarian cancer. Consequently, our findings consistently indicated that circ_100395 might be a crucial molecular target for ovarian cancer patients.

To elucidate the biological function of circ_100395 in ovarian cancer cells, we performed the CCK-8 and Transwell assays to detecte the proliferation and metastasis abilities, by upregulating circ_100395 in the SKOV3 and ES-2 ovarian cancer cells. We found that overexpression of circ_100395 could significantly suppress cell proliferation. Furthermore, our study also showed that overexpression of circ_100395 could dramatically reduce migration and invasion numbers of ovarian cancer cells.

Ovarian cancers are surface epithelial-stromal tumors, which means ovarian epithelial carcinoma is highly related with EMT signaling pathway^14^. In fact, EMT is a significant biological process that the normal epithelial cells were transformed into the mesenchymal cell with enhanced migratory and invasive properties. Emerging evidences demonstrated that the EMT was an important signaling pathway for activating the tumor migrated ability, but its function would be suppressed by circRNAs^20^-^22^. For example, Chen et al. revealed that circPRMT5 could activate the EMT signaling pathway, then promote the cell migrated ability in Urothelial Carcinoma of Bladder^23^. In the current study, we found that overexpression of circ_100395 led to the upregulation of the epithelial marker (E-cadherin) and downregulation of mesenchymal markers (N-cadherin and Snail). Therefore, we believe that circ_100395 could inhibit proliferation and metastasis of ovarian cancer cells, by activating EMT process.

Emerging evidence has shown that circRNAs could regulate cancer cell proliferation by inhibiting the downstream microRNAs targets, and this is called “cavernous mechanism”. For example, Wang et al. indicated that circ_0027599 suppresses the gastric cancer progression by sponging miR-101-3p^24^. Chen et al. reported that circ_100395 regulated miR-1228/TCF21 pathway to inhibit lung cancer progression^17^. In order to further explore the downstream regulation of circ_100395, a bioinformatics software (Starbase) was used to predict that miR-1228 might be the downstream target miRNA of circ_100395. From our assays, the expression level of miR-1228 was negatively correlated with the expression of circ_100395 in ovarian cancer tissues. In addition, circ_100395 could directly bind to miR-1228, while circ_100395-overexpressed ovarian cancer cells were more likely to have lower expression of miR-1228. Consequently, our results indicated that miR-1228 did being highly expressed in patients with ovarian cancer, and its negative relationship with circ_100395 indicated that miR-1228 might be the downstream of circ_100395. To test this hypothesis, another validation cohort containing 30 patients from another center was also applied. The results consistently showed that miR-1228 was significantly overexpressed in ovarian cancer tissues, while expression level of miR-1228 was negatively correlated with the expression of circ_100395. Moreover, the results showed that miR-1228 could reverse the inhibited ES-2 cell proliferation, metastasis and EMT process induced by the overexpression of circ_100395. Consequently, circ_100395 could play its role by sponging to miR-1228 in ovarian cancer.

Numerous studies have reported that miR-1228 is closely associated with the occurrence and development of malignant tumors. For example, Lin et al. indicated that miR-1228 could down-regulate the SCAI expression, then facilitate the cell proliferation and invasion in breast cancer or other caners^25^, ^26^. However, the function and its related mechanism of miR-1228 for ovarian cancer still unclear. In this study, the results of bioinformatics analysis indicated that p53 would be the target down-stream gene of miR-1228, and which their relationship was also confirmed by dual-luciferase reporter assay. Together, these results consistently showed that the circ_100395 suppressed the expression of miR-1228, then promoted the p53 protein expression and inhibited ovarian cancer progression. Previous studies had shown that p53 protein was involved in tumor cell proliferation, migration and invasion abilities. Furthermore, Zhang et al. have found that miR-1228 promoted the proliferation and metastasis of hepatoma cells through a p53 forward feedback loop. Consistent with the previous results, our study showed that p53 was downregulated in ovarian cancer, and miR-1228 promoted tumor process by inhibiting the expression of p53 in ovarian cancer.

## Conclusion

In summary, the expression level of circ_100395 is found to be downregulated in ovarian cancer. Overexpression of circ_100395 could suppress proliferation, migration and invasion of ovarian cancer cells through modulating miR-1228/p53/EMT axis. Therefore, circ_100395 may be considered as a cancer-suppressor gene and might serve as a promising biomarker for the treatment of ovarian cancer. However, there are also some limitations for this study, as the clinical samples were too litter to completely evaluate the sensitivity and specificity of circRNA_100395 in prognosis prediction of ovarian cancer patients. Therefore, we will increase the tissues sample size from other center to completely identify the biological function of circRNA_100395 in the following studies.

## Supplementary Material

Supplementary figures and tables.

## Figures and Tables

**Table 1:** Sequences of oligomers and primers used in the present research

Name	Sequence (5'-3')
Circ_100395-F'	AGT GAT GTG GCC CCT ACA AG
Circ_100395-R'	CCA CTG GAG ACC ACT GGT TG
p53-F'	CGC TAC CTC CTG AGA TTG GA
p53-R'	GGT TGC TGT GCA GGA CAA G
β-actin-F'	TCC GCA AAG ACC TGT ACG A
β-actin-R'	GTA CTT GCG CTC AGG AGG AG
miR-1228-RT	GTCGTATCCAGTGCAGGGTCCGAGGTATTCGCACTGGATACGACCACACA
miR-1228-F'	AGTGGGCGGGGGCAGG
miR-1228-R'	AGTGCAGGGTCCGAGGTATT

**Table 2:** Correlation analyses between relative circ_100395 expression and clinicopathological parameters of patients with ovarian cancer

Clinicopathological parameters		circ_100395		χ^2^	*P*
		Low	High		
Age (years)	<60	12 (46.2%)	14 (53.8%)	0.271	0.602
	≥60	18 (52.9%)	16 (47.1%)		
Lymphatic metastasis	Negative	9 (31.0%)	20 (69.0%)	8.076	0.004
	Positive	21 (67.7%)	10 (32.3%)		
CA-125 (U/mL)	<500	15 (44.1%)	19 (55.9%)	1.086	0.297
	≥500	15 (57.7%)	11 (42.3%)		
Distant metastasis	Negative	22 (44.0%)	28 (56.0%)	4.320	0.038
	Positive	8 (80.0%)	2 (20.0%)		
FIGO	I+II	7 (21.9%)	25 (78.1%)	21.696	0.000
	III+IV	23 (82.1%)	5 (17.9%)		

## References

[1] Bray F, Ferlay J, Soerjomataram I, Siegel RL, Torre LA, Jemal A (2018). Global cancer statistics 2018: GLOBOCAN estimates of incidence and mortality worldwide for 36 cancers in 185 countries. CA: a cancer journal for clinicians.

[2] Torre LA, Bray F, Siegel RL, Ferlay J, Lortet-Tieulent J, Jemal A (2015). Global cancer statistics, 2012. CA: a cancer journal for clinicians.

[3] Llueca A, Serra A, Maiocchi K, Delgado K, Jativa R, Gomez L (2019). Predictive model for major complications after extensive abdominal surgery in primary advanced ovarian cancer. International journal of women's health.

[4] Gao XP, Liu YH, Liu ZY, Wang LJ, Jing CX, Zhu S (2019). Pretreatment lymphocyte-to-monocyte ratio as a predictor of survival among patients with ovarian cancer: a meta-analysis. Cancer management and research.

[5] Wang BG, Xu Q, Lv Z, Fang XX, Ding HX, Wen J (2018). Association of twelve polymorphisms in three onco-lncRNA genes with hepatocellular cancer risk and prognosis: A case-control study. World journal of gastroenterology.

[6] Qu D, Sun WW, Li L, Ma L, Sun L, Jin X (2019). Long noncoding RNA MALAT1 releases epigenetic silencing of HIV-1 replication by displacing the polycomb repressive complex 2 from binding to the LTR promoter. Nucleic acids research.

[7] Li M, Wang J, Liu D, Huang H (2018). Highthroughput sequencing reveals differentially expressed lncRNAs and circRNAs, and their associated functional network, in human hypertrophic scars. Molecular medicine reports.

[8] Saleembhasha A, Mishra S (2018). Novel molecules lncRNAs, tRFs and circRNAs deciphered from next-generation sequencing/RNA sequencing: computational databases and tools. Briefings in functional genomics.

[9] Li Y, Chen B, Huang S (2018). Identification of circRNAs for miRNA Targets by Argonaute2 RNA Immunoprecipitation and Luciferase Screening Assays. Methods in molecular biology (Clifton, NJ).

[10] Militello G, Weirick T, John D, Doring C, Dimmeler S, Uchida S (2017). Screening and validation of lncRNAs and circRNAs as miRNA sponges. Briefings in bioinformatics.

[11] Mishra S, Verma SS, Rai V, Awasthee N, Chava S, Hui KM Long non-coding RNAs are emerging targets of phytochemicals for cancer and other chronic diseases. 2019; 76: 1947-66.

[12] Esfandi F, Taheri M Expression of long non-coding RNAs (lncRNAs) has been dysregulated in non-small cell lung cancer tissues. 2019; 19: 222.

[13] Ning L, Long B, Zhang W, Yu M, Wang S, Cao D (2018). Circular RNA profiling reveals circEXOC6B and circN4BP2L2 as novel prognostic biomarkers in epithelial ovarian cancer. International journal of oncology.

[14] Chen Q, Zhang J, He Y, Wang Y (2018). hsa_circ_0061140 Knockdown Reverses FOXM1-Mediated Cell Growth and Metastasis in Ovarian Cancer through miR-370 Sponge Activity. Molecular therapy Nucleic acids.

[15] Luo L, Gao Y, Sun X (2018). Circ-ITCH correlates with small tumor size, decreased FIGO stage and prolonged overall survival, and it inhibits cells proliferation while promotes cells apoptosis in epithelial ovarian cancer. Cancer biomarkers: section A of Disease markers.

[16] Wang ST, Liu LB, Li XM, Wang YF, Xie PJ, Li Q (2019). Circ-ITCH regulates triple-negative breast cancer progression through the Wnt/beta-catenin pathway. Neoplasma.

[17] Chen D, Ma W, Ke Z, Xie F (2018). CircRNA hsa_circ_100395 regulates miR-1228/TCF21 pathway to inhibit lung cancer progression. Cell cycle (Georgetown, Tex).

[18] Liu H, Xue L, Song C, Liu F, Jiang T, Yang X (2018). Overexpression of circular RNA circ_001569 indicates poor prognosis in hepatocellular carcinoma and promotes cell growth and metastasis by sponging miR-411-5p and miR-432-5p. Biochemical and biophysical research communications.

[19] Rong D, Dong C, Fu K, Wang H, Tang W, Cao H (2018). Upregulation of circ_0066444 promotes the proliferation, invasion, and migration of gastric cancer cells. OncoTargets and therapy.

[20] Singh M, Yelle N, Venugopal C, Singh SK (2018). EMT: Mechanisms and therapeutic implications. Pharmacology & therapeutics.

[21] Jie XX, Zhang XY, Xu CJ (2017). Epithelial-to-mesenchymal transition, circulating tumor cells and cancer metastasis: Mechanisms and clinical applications. Oncotarget.

[22] Yousefi M, Bahrami T, Salmaninejad A, Nosrati R, Ghaffari P, Ghaffari SH (2017). Lung cancer-associated brain metastasis: Molecular mechanisms and therapeutic options. Cellular oncology (Dordrecht).

[23] Chen X, Chen RX, Wei WS, Li YH, Feng ZH, Tan L (2018). PRMT5 Circular RNA Promotes Metastasis of Urothelial Carcinoma of the Bladder through Sponging miR-30c to Induce Epithelial-Mesenchymal Transition. Clinical cancer research: an official journal of the American Association for Cancer Research.

[24] Wang L, Shen J, Jiang Y (2018). Circ_0027599/PHDLA1 suppresses gastric cancer progression by sponging miR-101-3p.1. Cell & bioscience.

[25] Lin L, Liu D, Liang H, Xue L, Su C, Liu M (2015). MiR-1228 promotes breast cancer cell growth and metastasis through targeting SCAI protein. International journal of clinical and experimental pathology.

[26] Zhang Y, Dai J, Deng H, Wan H, Liu M, Wang J (2015). miR-1228 promotes the proliferation and metastasis of hepatoma cells through a p53 forward feedback loop. British journal of cancer.

