# CircRNA_100395 inhibits cell proliferation and metastasis in ovarian cancer via regulating miR-1228/p53/epithelial-mesenchymal transition (EMT) axis: Erratum

**DOI:** 10.7150/jca.93299

**Published:** 2024-02-01

**Authors:** Xian Li, Shuihua Lin, Zhifeng Mo, Jinxing Jiang, Haifeng Tang, Cailin Wu, Jian Song

**Affiliations:** 1 Department of Obstetrics and Gynaecology, The University of Hong Kong - Shenzhen Hospital, Shenzhen, Guangdong, China.; 2 Department of Medical Imaging, The University of Hong Kong - Shenzhen Hospital, Shenzhen, Guangdong, China.; 3 Department of Emergency & Disaster Medical Center, The Seventh Affiliated Hospital, Sun Yat-sen University, Shenzhen, Guangdong, China.; 4 Cytotherapy Laboratory, Shenzhen People's Hospital, The Second Clinical Medical College of Jinan University, Shenzhen, Guangdong, China.; 5 Department of Obstetrics and Gynaecology, The University of Hong Kong - Shenzhen Hospital, Shenzhen, Guangdong, China.; 6 Department of Obstetrics and Gynaecology, The University of Hong Kong - Shenzhen Hospital, Shenzhen, Guangdong, China.; 7 Department of Obstetrics and Gynaecology, The University of Hong Kong - Shenzhen Hospital, Shenzhen, Guangdong, China.

In our paper, there were errors in Fig. 6C. A comprehensive review of the original data associated with the questionable images was conducted. These errors occurred when other users mistakenly saved their images to our folder on the shared computer. Regrettably, we inserted the wrong images into Fig. 6C, with no intention of data manipulation. The correct Fig. 6 is provided below. The robustness of the research conclusions in the figures has not been compromised by these errors. Below, please find the correction details. We are truly sorry for our careless mistakes and apologize for any confusion they may have caused readers. Measures have been taken to ensure that such errors do not recur in the future.

## Figures and Tables

**Figure 6:**
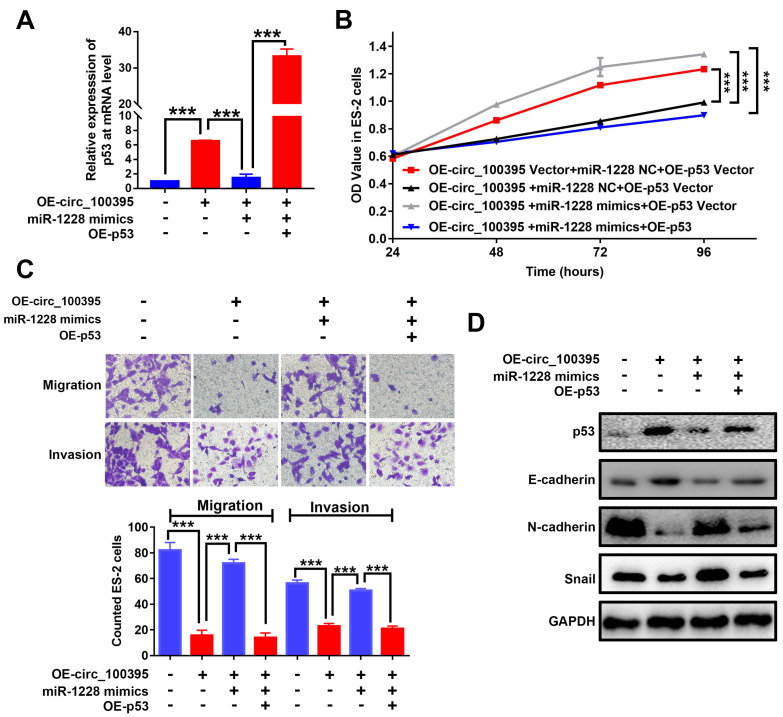
circ_100395 suppresses the proliferation, migration, invasion and EMT in ovarian cancer cells, by sponging to miR-1228 and regulating p53 in ovarian cancer. (A) The expression level of p53 in circ_100395 or miR-1228 or p53 up-regulated ovarian cancer cells. (B) The overexpression of circ_100395 or miR-1228 or p53 regulate the proliferation of ES-2 cells in vitro using CCK-8 assay. (C) The overexpression of circ_100395 or miR-1228 or p53 significantly regulated the number of migrated and invaded cells in ES-2 cells, measured by Transwell assay. (D) Overexpression of circ_100395 or miR-1228 or p53 dramatically changed the expression of the epithelial marker, E-cadherin, while the mesenchymal markers, N-cadherin and Snail were contrarily regulated. *** *P* < 0.001.

