# Supplementary material for: CircRNA_100395 inhibits cell proliferation and metastasis in ovarian cancer via regulating miR-1228/p53/epithelial-mesenchymal transition (EMT) axis
Source: J Cancer. 2020 Jan 1;11(3):599–609. doi: 10.7150/jca.35041 (PMC6959051; doi:10.7150/jca.35041)
Supplement: Supplementary file 1 — Supplementary figures and tables. [file jcav11p0599s1.pdf]

Supplementary files

CircRNA_100395 inhibits cell proliferation and metastasis in ovarian cancer via regulating miR-1228/p53/epithelial-mesenchymal transition (EMT) axis

Author name Xian Li[*,#,1], Shuihua Lin*[,2] Zhifeng Mo[3], Jinxing Jiang[4], Haifeng Tang[5], Cailin Wu[6], Jian Song[7]

Results

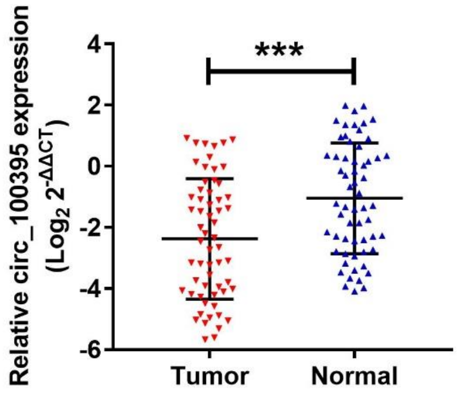

Figure S1. circ_100395 was significantly down-expressed in ovarian cancer tissues than that in adjacent normal tissues evaluated by RT-qPCR.

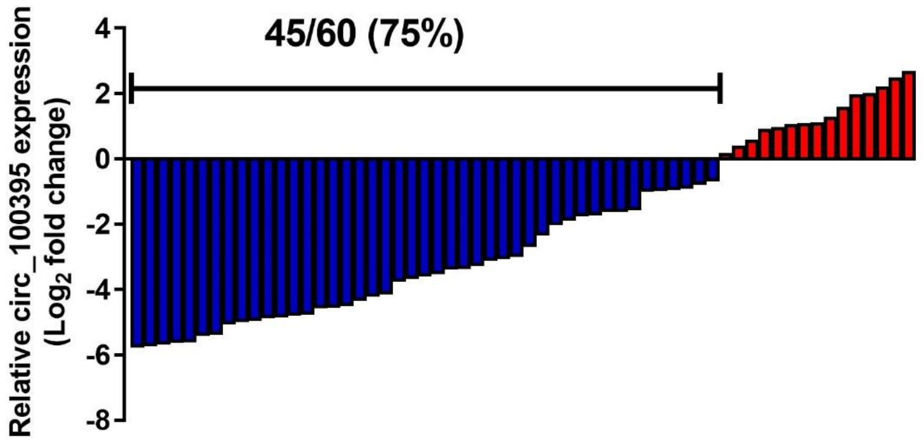

Figure S2 The relative circ_100395 expression was down-regulated in 75% (45/60) patients with ovarian cancer revealed as the form of Log2 (T/N).

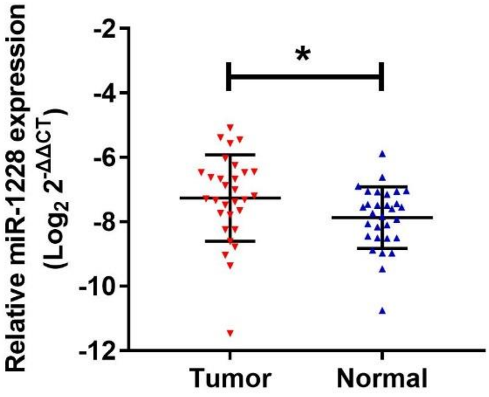

Figure S3. miR-1228 was significantly overexpressed in ovarian cancer tissues than that in adjacent normal tissues evaluated by RT-qPCR.

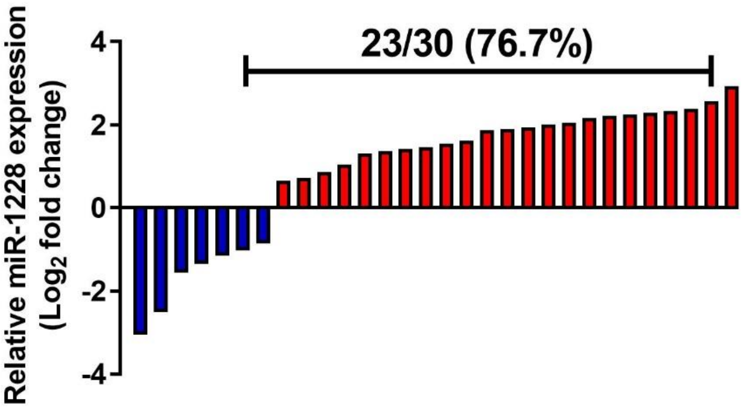

Figure S4. The relative miR-1228 expression was up-regulated in 76.7% (23/30) patients with ovarian cancer revealed as the form of Log2 (T/N).

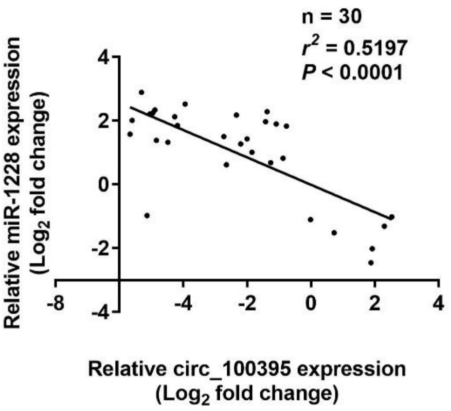

Figure S5. Correlation between circ_100395 and miR-1228 at mRNA levels in 30 paired human ovarian cancer tissues.
